# Multi-person dance tiered posture recognition with cross progressive multi-resolution representation integration

**DOI:** 10.1371/journal.pone.0300837

**Published:** 2024-06-13

**Authors:** Huizhu Kao

**Affiliations:** School of Music, Weifang University, Weifang, Shandong, China; Opole University of Technology: Politechnika Opolska, POLAND

## Abstract

Recognizing postures in multi-person dance scenarios presents challenges due to mutual body part obstruction and varying distortions across different dance actions. These challenges include differences in proximity and size, demanding precision in capturing fine details to convey action expressiveness. Robustness in recognition becomes crucial in complex real-world environments. To tackle these issues, our study introduces a novel approach, i.e., Multi-Person Dance Tiered Posture Recognition with Cross Progressive Multi-Resolution Representation Integration (CPMRI) and Tiered Posture Recognition (TPR) modules. The CPMRI module seamlessly merges high-level features, rich in semantic information, with low-level features that provide precise spatial details. Leveraging a cross progressive approach, it retains semantic understanding while enhancing spatial precision, bolstering the network’s feature representation capabilities. Through innovative feature concatenation techniques, it efficiently blends high-resolution and low-resolution features, forming a comprehensive multi-resolution representation. This approach significantly improves posture recognition robustness, especially in intricate dance postures involving scale variations. The TPR module classifies body key points into core torso joints and extremity joints based on distinct distortion characteristics. Employing a three-tier tiered network, it progressively refines posture recognition. By addressing the optimal matching problem between torso and extremity joints, the module ensures accurate connections, refining the precision of body key point locations. Experimental evaluations against state-of-the-art methods using MSCOCO2017 and a custom Chinese dance dataset validate our approach’s effectiveness. Evaluation metrics including Object Keypoint Similarity (OKS)-based Average Precision (AP), mean Average Precision (mAP), and Average Recall (AR) underscore the efficacy of the proposed method.

## 1. Introduction

Dance is one of the essential forms of cultural expression. In a typical dance class, there are often many students, making it challenging for teachers to accurately assess students’ real-time mastery of dance actions and emotional expressions Therefore, applying information technology to estimate the teaching status of dancers can significantly enhance the implementation of tailored teaching methods. As technology and culture become increasingly integrated, the recognition of posture in dance images is becoming an important application area for computer vision technology. It has a wide range of applications, including correcting the actions of professional dancers, self-guided dance instruction, sports analysis, competition judging, entertainment, game design, augmented reality (AR), virtual reality (VR), and various human-computer interaction scenarios.

Currently, there are two main categories of multi-person posture recognition methods: top-down and bottom-up. In the top-down approach, the image is first processed by an object detector to locate human bounding boxes. Then, the single-person posture recognition model is performed on each detected bounding box to estimate key points. Finally, these key points are connected to form the posture recognition results. Chen et al. [1] proposed the CPN (Cascaded Pyramid Network) method, which utilizes feature pyramids and RefineNet to recognize key points. Fang et al. [2] introduced the RMPE (Regional Multi-person Pose Estimation) method for posture recognition. Newell et al. [3] proposed the Hourglass network to combine features at different scales. Sun et al. [4] introduced the HRNet (High-Resolution Network), which uses high-resolution features for human posture recognition. On the other hand, bottom-up methods are divided into key point detection and clustering. These approaches use single-person posture recognition algorithms to detect all key points in the image and then cluster the keypoints of different individuals together to perform multi-person posture recognition. Typical representatives of this category include OpenPose [5], Deepcut [6], HigherHRNet [7], and Pifpaf [8].

Each of these two categories has its own advantages and disadvantages. Top-down methods break down posture recognition into two steps: object detection and single-person posture recognition, which results in higher accuracy due to relying on robust object detection and posture recognition algorithms. However, they are sensitive to the quality of object detection bounding boxes, and even state-of-the-art (SOTA) object detectors may introduce errors in terms of redundant bounding boxes, missed detections, or false positives. In contrast, bottom-up methods are faster as they do not rely on object detectors for bounding box detection. However, they face challenges when aggregating key points in the presence of obstructions, and when individuals are in close proximity, it can lead to ambiguities in clustering the key points, resulting in lower posture recognition accuracy. In addition, existing human posture recognition methods primarily target traditional datasets with simple poses such as standing or walking. However, dance actions are complex, diverse, and highly continuous, often involving strong obstructions, lighting changes, and variations in camera angles. These factors increase the difficulty of accurately estimating dance actions using traditional methods.

**Fig 1:**
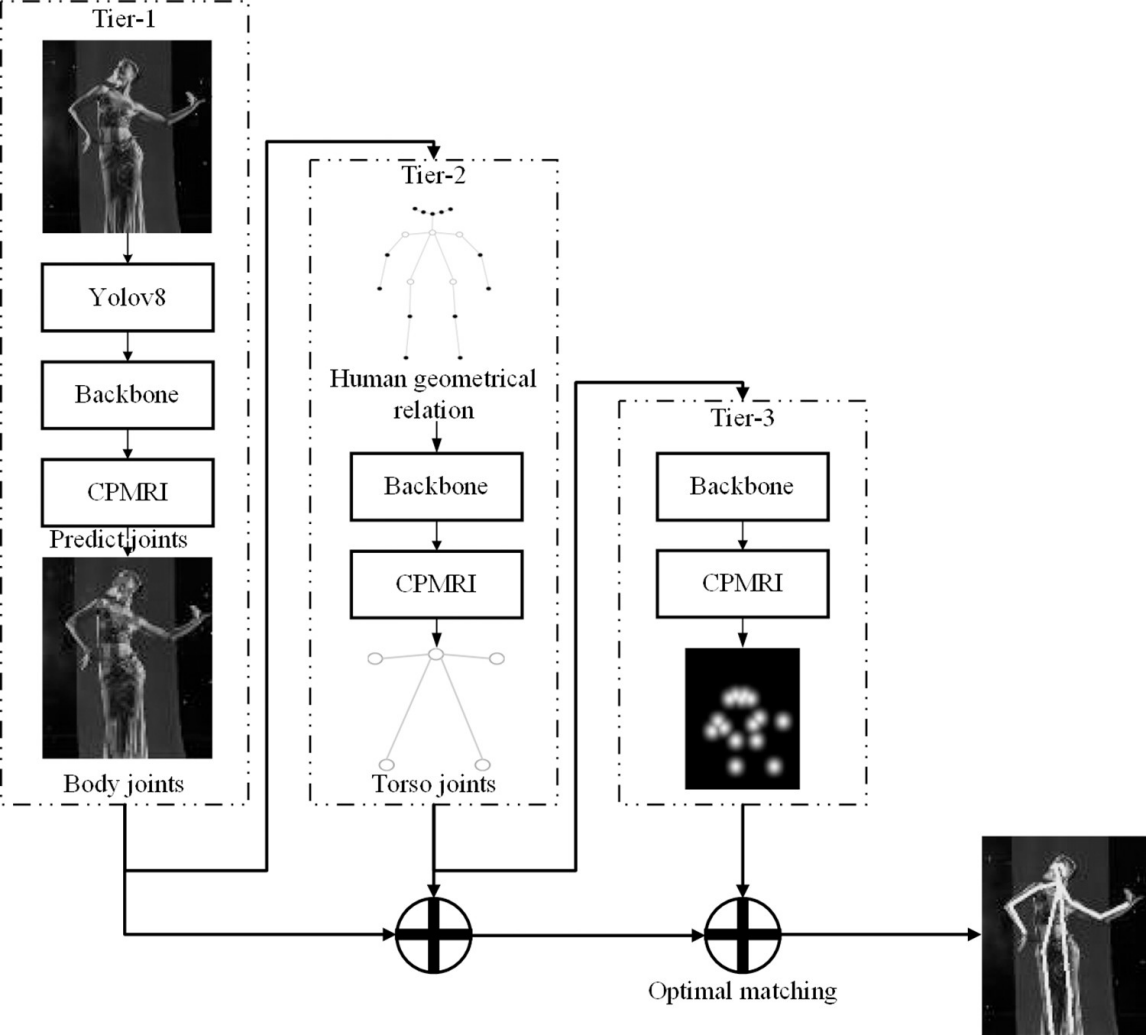
Multi-person dance tiered posture recognition with cross progressive multi-resolution representation integration.

To address these challenges, we propose a multi-person dance tiered posture recognition with cross progressive multi-resolution representation integration, as shown in Fig 1. The algorithm employs a top-down framework and begins with YOLOv8 for detecting dancers’ bounding boxes. To address the issue of rapidly changing scales in dance pose skeleton key points, it uses HRNet as the backbone network and introduces a Cross Progressive Multi-resolution Representation Integration (CPMRI) module. The CPMRI module fuses high and low-level multi-scale features to improve the robustness of posture recognition in response to scale variations. Furthermore, this paper addresses issues related to severe distortion and obstruction in dance poses by designing a Tiered Posture Recognition (TPR) module based on the geometrical relations between key points, enabling tiered key point estimation for accurate dancer key point positions. Finally, the proposed method’s effectiveness is verified on both public and custom dance datasets.

In the field of multi-person pose recognition, our contributions can be summarized as follows:

1) Classification of multi-person pose recognition methods: In the related work section, we have categorized existing multi-person pose recognition techniques into different classes, including top-down, bottom-up, and other approaches. This classification provides a systematic overview that is valuable for researchers and practitioners in this domain.

2) Application of YOLOv8 for dance posture recognition: We have employed YOLOv8, a state-of-the-art model known for its balance between accuracy and efficiency, as the human detector within the top-down framework. This marks the first instance of YOLOv8 being used specifically for dance posture recognition.

3) Development of CPMRI module: We propose the CPMRI module, a novel approach for integrating multi-resolution features. This module fuses high-level semantic features with low-level spatial details, resulting in enhanced feature representation for pose recognition.

4) Creation of TPR module: The TPR module is designed to enhance the recognition of dancer’s body key points by considering the geometrical relations between them. This module improves pose recognition accuracy through structured partitioning and matching processes.

5) Validation and performance improvement: The proposed method has been validated on both open and custom datasets, using various evaluation metrics. The results show significant improvements in the robustness and accuracy of multi-person dance pose recognition.

## 2. Related works

Human posture recognition is a fundamental component and a hot topic in computer vision research, serving as the basis for tasks such as action recognition, human-computer interaction, and pose tracking [9]. It involves locating and identifying key points or body parts on an individual, focusing on the problem of finding body parts’ positions. By locating key points in the skeletal model of the human body from images or videos, it enables pose analysis or the search for specific postures or actions within the target object based on the relationships between key points [10].

As a critical foundation of human posture recognition tasks, human body modeling significantly influences the final outcome of posture recognition [11]. Human body models used in posture recognition can be categorized into three types: silhouette-based, skeleton-based, and volumetric-based. Silhouette-based and skeleton-based models are typically used in two-dimensional human posture recognition tasks, while volumetric-based models are commonly employed in three-dimensional human posture recognition tasks. Silhouette-based models provide coarse width and contour information of extremities and the torso and were widely used in early posture recognition tasks. Skeleton-based human key point models represent the human pose graphically, and the number of key points can be flexibly chosen based on the task and dataset requirements. Common key point counts include 9, 14, 15, 16, 17, 18, 25, and 33.

**Fig 2:**
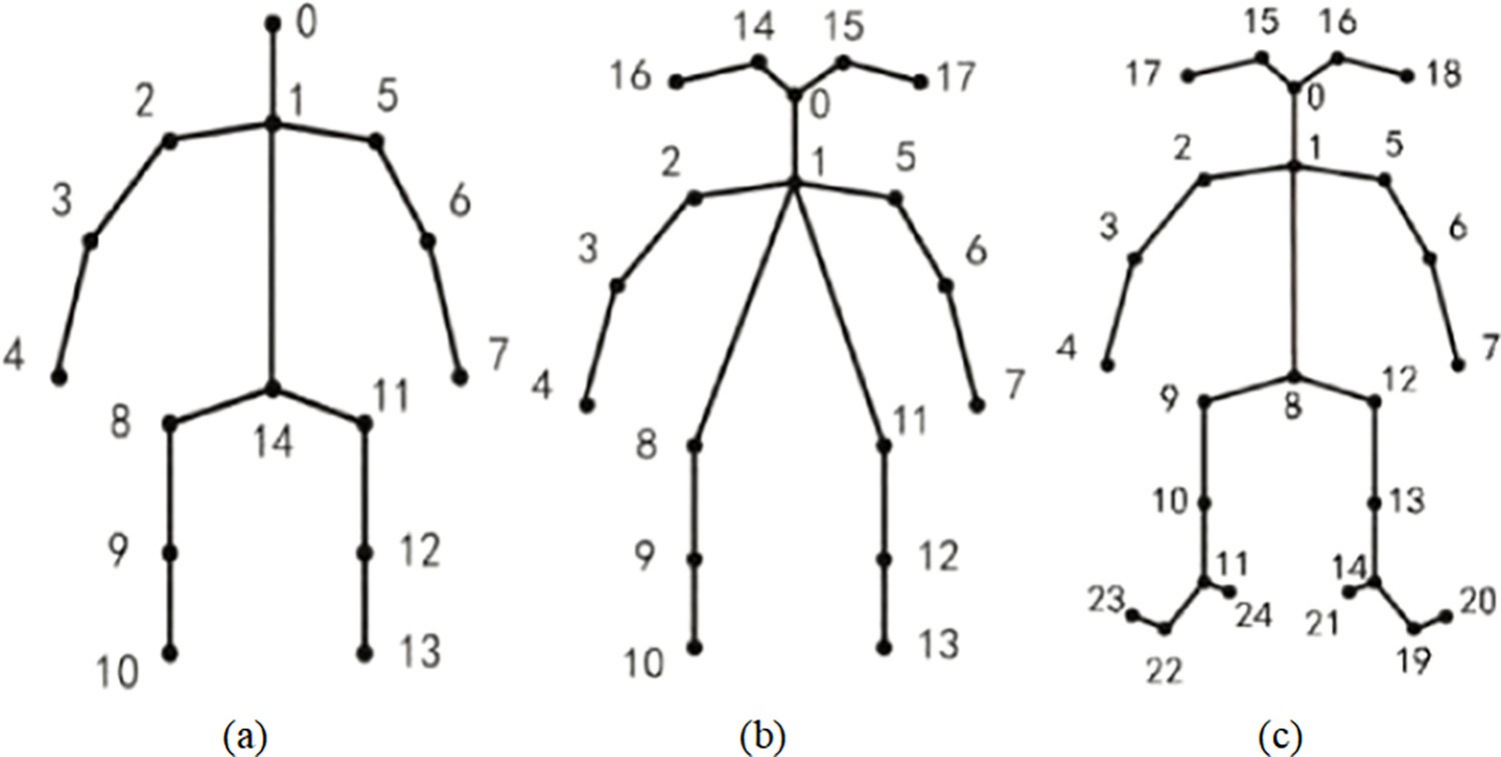
Human skeleton keypoints model. (a) 15 key points. (b) 18 key points. (c) 25 key points.

Fig 2 shows 15-point, 18-point, and 25-point human body skeleton key point models, with the 18-point model offering more refinement for head key points and the 25-point model including further refinement for foot keypoints. Skeletal models include correlated key points (such as right shoulder, right elbow, and right hand), where each coordinate corresponds to a key point in the human skeleton. It’s worth noting that human body models for 2D and 3D posture recognition have their own advantages and disadvantages. Volumetric-based models can more accurately describe human pose and spatial location, while silhouette and skeleton-based models have lower computational requirements and are suitable for scenarios with real-time demands.

In response to the real-time requirements of practical applications, some methods have emerged that project 3D human body models into 2D representations. These methods, such as using character modeling and animation models to outline the human silhouette to express non-rigid distortions of articulated limbs [12], or projecting character models and animation models into 2D-constructed outlines that can be further trained as deformable structures [13], have advanced the development of 2D human pose recognition.

Traditional posture recognition methods can be divided into two stages: object detection and pose recognition. In the object detection stage, methods based on segmentation and matching, gradient information, or statistical learning are typically used. However, these methods face challenges in separating the object from the background in complex scenes, are susceptible to the influence of prior information, and require significant time and effort to construct training sample libraries and classifiers [14]. In the pose estimation stage, both model-based and non-model-based methods are commonly employed, but they often encounter issues such as the propagation of errors from the object detection stage, heavy reliance on artificially imposed constraints, and low efficiency [15].

In recent years, deep learning-based methods for human posture recognition have been proposed, greatly alleviating the drawbacks of traditional methods, which relied heavily on manually annotated features and had low accuracy [16]. Through algorithm and network model improvements, deep learning-based human posture recognition methods have demonstrated increasingly promising experimental results and have gradually become the mainstream approach in this field.

Compared to single-person posture recognition, multi-person posture recognition faces additional challenges, including issues related to the correct detection of key points due to crowded and occluded scenes and the difficulty in establishing effective connections between correlated key points [17]. Multi-person posture recognition aims to recognize and locate key points for all individuals in an image, making it a fundamental research topic for various visual applications such as human action recognition and human-computer interaction.

**Fig 3:**
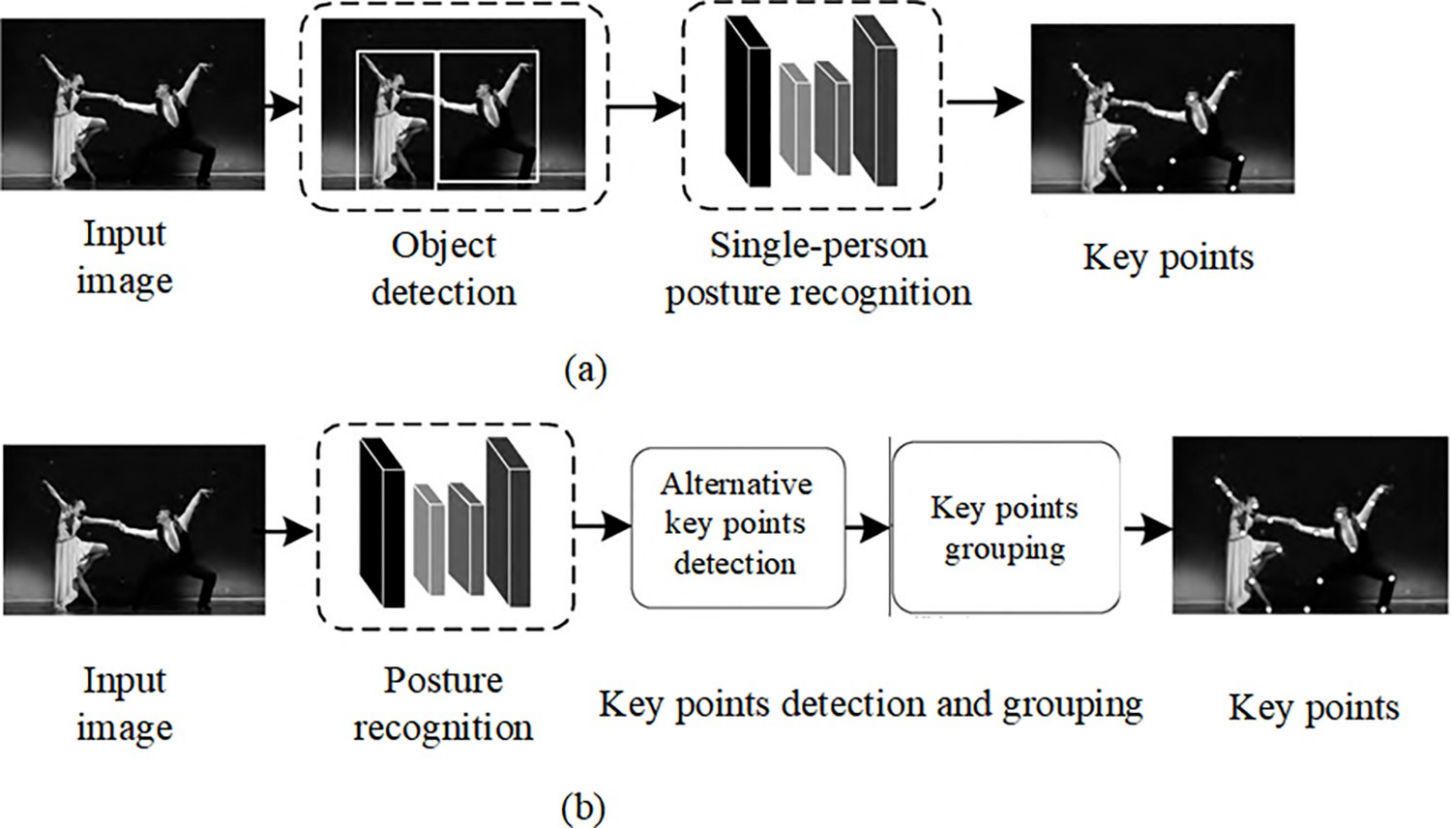
Multi-person posture recognition process. (a) Top-down. (b) Bottom-up.

The models for multi-person posture recognition can be categorized into top-down, bottom-up, and a few other methods that do not belong to these two categories. Top-down methods first locate individuals using detectors and then predict key point positions using single-person pose estimation methods. Bottom-up methods first identify identity-agnostic semantic entities (consisting of individual key points and body part segmentation labels) and then group them into human instances. Generally, top-down networks offer higher precision, while bottom-up networks provide better real-time performance. The multi-person posture recognition process is illustrated in Fig 3.

### 2.1 Top-down methods

In the top-down pipeline as shown in Fig 3(A), it consists of a detector and a single-person posture recognizer. These components are responsible for acquiring human bounding boxes and predicting the key point positions within those boxes. Several works have focused on the design and improvement of modules within the posture recognition network. Xiao et al. [18] introduced a simple and effective structure into the ResNet backbone network, adding deconvolution layers to generate high-resolution heatmaps. To better locate key point positions, Wang et al. [19] introduced a two-stage model based on graphs, known as Graph-PCNN. It includes a localization subnetwork for obtaining rough key point positions and a graphical pose refinement module for precise key point localization. Cai et al. [20] introduced a multi-level network with Residual Step Networks (RSN) modules for learning fine local representations through effective internal feature fusion strategies. In situations with multiple people in a single image, body part obstruction and overlap inevitably occur. Therefore, in the top-down methods, human detection and identification have become challenging. To address this, Iqbal et al. [21] constructed a joint detector based on Faster R-CNN to estimate joint candidates. They constructed a fully connected graph of candidate key points detected in the image, then used integer linear programming to solve the association problem between joints and individuals, even in cases of severe obstruction. Papandreou et al. [22] proposed a two-stage architecture, featuring a faster R-CNN human detector to create bounding boxes for candidate individuals and a key point estimator that predicts key point positions using a form of heatmap offset aggregation. This method performs well in scenarios with obstruction and clutter. To mitigate obstruction issues, Chen et al. [1] introduced a Cascaded Pyramid Network (CPN) with two parts: GlobalNet (a feature pyramid network for predicting occluded key points) and RefineNet (a network that combines features from all levels of GlobalNet with key point mining losses). Experimental results show that CPN excels in predicting occluded key points. Su et al. [23] designed two modules, the Channel Shuffle Module (CSM) and the Spatial Channel-Wise Attention Residual Bottleneck (SCARB), to enhance channel-wise and spatial information for improved multi-person posture recognition in occluded scenes. Qiu et al. [24] proposed a novel OPEC network framework, introducing an image-guided progressive GCN module that provides a comprehensive understanding of image context, contextual cues, and pose structure, thereby estimating occluded joints from an inferential perspective. Rafi et al. [25] introduced a key point correspondence framework that uses temporal information from the previous frame in occluded scenarios to recover missing poses. They employed self-supervised training to enhance posture recognition results on sparsely annotated video datasets. Sheu et al. [26] developed a novel approach tailored for single-person pose estimation and action recognition. This framework is particularly well-suited for fitness and mobility applications. It starts with a base network that generates an initial pose estimate, which is then refined through the Intensive Feature Consistency (IFC) network. The IFC network applies high-level constraints to both global body intensity correction and local body part adjustments, focusing on maintaining long-term consistency in joint movements. This approach significantly enhances posture recognition accuracy, as demonstrated through improvements on benchmark datasets. The IFC network not only achieves high precision in keypoint localization but also maintains real-time processing capability on CPU platforms, showcasing a notable advancement in the field of posture recognition. Zhang et al. [27] employed a adversarial method to incorporate prior knowledge of human pose configurations into the training process of their model. They utilized a hierarchy of fully convolutional networks, acting as a discriminator, to differentiate between authentic and artificial poses. This innovative approach encourages the pose estimation network to generate poses that are perceived by the discriminator as genuine. Such a strategy proves particularly effective in complex scenarios, where the pose network is driven to produce estimations that even the discriminator cannot distinguish from real poses. This methodology not only enhances the accuracy of pose estimation but also addresses the challenge of discerning between real and fabricated pose data, a common obstacle in the field of human pose estimation.

More recently, transformer-based approaches have gained attention because the attention modules within transformers can capture long-range dependencies and global evidence for predicting key points, making them more powerful than CNNs (Convolutional Neural Networks). An early example of a transformer-based 2D human posture recognition model is TransPose, which uses attention layers to predict keypoints’ heatmaps and learns fine-grained evidence for human posture recognition in occluded scenes. Following TransPose, many existing CNN-based methods have excelled in visual representations. However, they lack the ability to explicitly learn the constraints between key points. Li et al. [28] developed a pure transformer model called TokenPose, which uses token representations to capture constraint clues and visual appearance relationships. Through this approach, TokenPose can explicitly capture appearance cues and constraint clues through self-attention interactions.

### 2.2 Bottom-up methods

Unlike the top-down method, the bottom-up method first involves the detection of human key points, extracting local features, and predicting candidate body joints, as shown in Fig 3(B). Subsequently, these joint candidates are grouped to build a pose representation using part association strategies. Pishchulin et al. [6] introduced a body part detector called DeepCut, based on Fast R-CNN. It combines labeled parts using Integer Linear Programming (ILP). However, DeepCut is computationally expensive. To address this, Insafutdinov et al. [29] introduced DeeperCut, a robust body part detector that introduced new image-conditioned pairwise terms between body parts, along with an incremental optimization strategy to improve speed and robustness. Later, Cao et al. [5] developed a detector named OpenPose, which is based on the CPM network. It predicts all candidate key point information with Part Affinity Fields (PAF), encoding limb positions and directions as 2D vector fields.

In the top-down approach, OpenPose improved detection speed to some extent. Osokin et al. [30] optimized OpenPose further and introduced Lightweight OpenPose. It utilized MobileNet v1 for feature extraction and reduced computational load through weight sharing. To address the issue of inadequate receptive fields, they adopted an optimized algorithm using dilated convolutions. Based on the OpenPose framework, Zhu et al. [31] improved the OpenPose structure by introducing a redundant PAF mechanism to address the shortcomings of the original PAF and enhance connections between joints in PAF, resulting in better performance than the baseline method. To enhance the performance of the OpenPose algorithm under conditions of low resolution or incomplete target acquisition, Kreiss et al. [8] introduced a method called PifPaf for multi-person posture recognition. This method uses Part Intensity Fields (PIF) to predict body part positions and PAF to associate body parts to determine individual complete human poses and grouping. The compound PAF encodes fine-grained information. In low-resolution and occluded scenes, this method outperforms previous OpenPose-based methods. Inspired by OpenPose, Newell et al. [32] introduced the concept of associative embedding, a novel approach to training supervised CNNs for detection and grouping tasks. The network simultaneously outputs detection and part assignments, making it embeddable in other network architectures. Following associative embedding, Jin et al. [33] introduced a new method called differentiable Hierarchical Graph Grouping (HGG) for learning graph grouping in a bottom-up multi-person posture recognition task.

Furthermore, HGG is easily embeddable into mainstream bottom-up methods. It takes human key point candidates as graph nodes and clusters key points in a multi-level graph neural network model. HGG modules are trainable end-to-end through key point detection networks and can monitor the grouping process in a hierarchical manner. Based on associative embedding and HRNet, Cheng et al. [7] introduced an extension of HRNet called High-Resolution Network (HigherHRNet). It learns scale-aware representations through a high-resolution feature pyramid and performs multi-resolution supervised training. During inference, HigherHRNet with multi-resolution heatmap aggregation effectively generates multi-resolution and higher-resolution heatmaps for more accurate human posture recognition. Yu et al. [34] proposed LiteHRNet, an efficient high-resolution network for human posture recognition. It introduced a lightweight unit called conditional channel weighting to replace costly pointwise (1×1) convolutions in the unordered block. Channel weighting has a linear complexity relative to the number of channels and lower time complexity than pointwise convolutions.

Bottom-up methods also incorporate multi-task structures. Papandreou et al. [35] introduced PersonLab, which combines posture recognition modules with human segmentation modules for key point detection and association. PersonLab comprises short-range offset (for refining heatmaps), mid-range offset (for predicting keypoints), and long-range offset (for grouping keypoints into instances). In addition, Kocabas et al. [36] proposed a multi-task learning model with a pose residual network called MultiPoseNet, capable of performing key point prediction, human detection, and semantic segmentation tasks.

Accurate regression of key point positions requires learning representations that focus on key point areas. Geng et al. [37] introduced a simple and effective method called decoupled key point regression. It employs adaptive convolutions via pixel-wise spatial transformers to activate pixels within key point regions and learn representations accordingly. Heatmap regression is a prevalent choice in today’s human posture recognition methods. Ground-truth heatmaps are typically constructed by covering all skeletal keypoints with 2D Gaussian kernels. The standard deviation of these kernels is fixed. However, for bottom-up methods that need to handle a wide range of human scale variations and label ambiguity, Luo et al. [38] introduced scale-adaptive heatmap regression, which adaptively adjusts the standard deviation for each keypoint. Due to high computational costs, it’s challenging to deploy state-of-the-art posture recognition models on resource-constrained edge devices. The high-resolution branches of HRNet are redundant for modes with low computation areas, and they can be removed to improve efficiency and performance. Inspired by this discovery, Wang et al. [39] designed LitePose, an efficient single-branch architecture for posture recognition and introduced two simple methods to enhance LitePose’s capability, including fusing deconvolution heads and large-kernel convolutions. Large-kernel convolutions significantly improve model capacity and receptive field, while maintaining lower computational costs. Li et al. [40] proposed the MRNet, focusing on the challenge posed by occlusion in monocular image-based posture recognition. This method introduces the concept of an occlusion level for human body parts, calculated based on the occlusion ratio and orientation of these parts. To adapt to different levels of visibility, MRNet establishes specific part detectors for each body part relative to its occlusion level. This innovation allows for improved accuracy in posture recognition by effectively addressing the common issue of body part occlusion in single-camera images. Cheng et al. [41] introduced a dual anatomical approach to address the challenge of scale variation in human pose estimation, particularly for small-scale individuals. Firstly, they propose multi-scale training as a means to enhance the network’s ability to handle variations in scale, coupled with single-scale testing to specifically improve performance on small-scale persons. Secondly, they innovated by introducing the concept of dual anatomical centers, focusing on the head and body as pivotal points for prediction. This allows for more accurate and reliable human pose predictions, especially beneficial for small-scale persons. This dual-pronged strategy marks a significant advancement in the field, providing a robust solution to the perennial issue of scale variation in human pose estimation tasks.

### 2.3 Other methods

In the realm of multi-person posture recognition, there exist a few methods that do not fit neatly into either the top-down or bottom-up categories. These methods can be categorized based on their characteristics into single-stage networks, top-down and bottom-up hybrid networks, and generative adversarial posture recognition networks. This section provides an introduction to some of these approaches, namely the Single-Stage Multi-Person Pose Machine (SPM), the typical hybrid network Hourglass, Deeply Learned Compositional Models (DLCM), and Generative Partition Networks (GPN).

SPM [42]: Addressing the resource-intensive and non-real-time nature of two-stage methods, SPM introduced a single-stage solution to human posture recognition. It offers a trade-off between speed and precision when compared to two-stage methods. SPM employs Structured Pose Representation (SPR) to link body position information with corresponding key point information. SPR encodes key point locations by considering robust and critical key points as central points. To tackle the long-distance offset issue between key points and central points caused by significant body actions, a layered SPR model is used. Additionally, the layered SPR model uses the short-distance positional information between closely located key points in adjacent layers to identify key point locations.

Single-stage and multi-stage bottom-up methods each have their advantages and drawbacks. Single-stage methods harness the end-to-end network’s ability to map input images to human poses, offering faster computation speeds compared to multi-stage methods. In contrast, multi-stage networks predict human poses in multiple stages and introduce intermediate supervision to address gradient vanishing issues, resulting in higher precision compared to single-stage methods.

2) Hourglass [3]: This method aims to address the challenge of processing and integrating features at all scales for capturing various spatial relationships related to the body. Hourglass proposes stacking multiple Hourglass modules with skip connections to form the network architecture. It employs plug-and-play supervision, which effectively conducts a bottom-up operation on the target individual before proceeding with the top-down operation. The bottom-up process downsizes the image from high resolution to low resolution, and the top-down process restores the image from low resolution to high resolution by combining features at multiple resolutions. It’s important to note that Hourglass is primarily used for single-person posture recognition, and for multi-person tasks, multiple instances of the image need to be cropped to ensure that the target individuals are centered in the images.

3) DLCM [43]: DLCM improves upon existing compositional models by utilizing Hourglass to obtain paired key point pose predictions. It offers a two-stage processing approach with top-down inference and bottom-up refinement, better describing complex compositional relationships between different body parts. DLCM is capable of resolving ambiguities present in bottom-up pose predictions, resulting in more precise posture recognitions.

4) GPN [44]: GPN addresses the issues of high complexity, low joint detection and recombination accuracy in previous multi-person posture recognition methods. It introduces a new generative partition strategy, creating partitions for multiple nodes from global candidate nodes and simultaneously inferring the joint positions for specific instances. GPN employs Hourglass as its backbone network and uses a joint detector and dense regressor for posture recognition. The joint detector sequentially examines all human entities in the input data, resulting in lower joint detection complexity and robustness against early detection errors compared to the top-down approach. The dense regressor produces human detection with key point partitions. GPN exhibits robustness against obstruction, overlap, distortion, and significant motion variations, and it offers notable advantages in terms of precision and speed in posture recognition.

5) Hybrid Parallel Network (HPnet) [45]: This hybrid model combines convolutional and transformer models to address the limitations of existing hybrid human posture recognition models, which often suffer from mutual conflicts between sequential self-attention and convolution modules. By parallelizing these modules, HPnet effectively leverages their complementary capabilities. The self-attention branch models long-range dependencies to enhance semantic representation, while the convolution branch focuses on high-precision localization. Additionally, a cross-branches attention module is introduced to further mitigate conflicts and synchronize features across both branches. HPnet shows remarkable performance on COCO validation and test-dev sets, achieving both high-precision keypoint localization and consistent overall performance, placing it on par with state-of-the-art human posture recognition models.

## 3. Materials and methods

In this paper, we introduce a novel multi-person dance posture recognition system that integrates cross progressive multi-resolution representations, as depicted in Fig 1. The system utilizes a top-down approach, commencing with the YOLOv8 algorithm for detecting dancers’ bounding boxes. To tackle the challenge of varying scales in dance pose skeletons, HRNet is employed as the backbone network, complemented by the CPMRI module. This module effectively fuses high and low-level multi-scale features, enhancing the robustness of posture recognition against scale variations. Additionally, the TPR module is introduced to address the issues of severe distortion and obstruction in dance poses. The TPR module, grounded in the geometrical relationships between key points, facilitates tiered key point estimation, thereby ensuring accurate localization of dancer key points. The following sections will provide a detailed introduction.

### 3.1 human body bounding box detection using YOLOV8

In this paper, an end-to-end algorithm is employed for human body detection, i.e., it utilizes the YOLOv8 detector [46] to extract bounding boxes around the dancers’ bodies. RGB images are fed into the YOLOv8 model to obtain corresponding bounding boxes for human body posture recognition.

The YOLOv8 model, released by Ultralytics in 2023, represents the latest model in the YOLO series. It is a one-stage object detection method designed to efficiently locate and identify objects in images. Object detection methods are primarily categorized into two types: two-stage detection methods, with Faster R-CNN [47] as a representative, and one-stage detection methods, with the YOLO series as an example. Two-stage detection algorithms involve two steps: the first step generates candidate boxes that may contain objects, followed by confidence analysis for each candidate box to select the best ones, and finally, classification and regression prediction for each candidate box. On the other hand, one-stage detection methods utilize CNNs to directly extract image features, generate feature vector maps, and then directly produce all anchor boxes for classification and regression prediction. Consequently, one-stage detection methods offer faster detection speeds and are more suitable for real-time applications.

**Fig 4:**
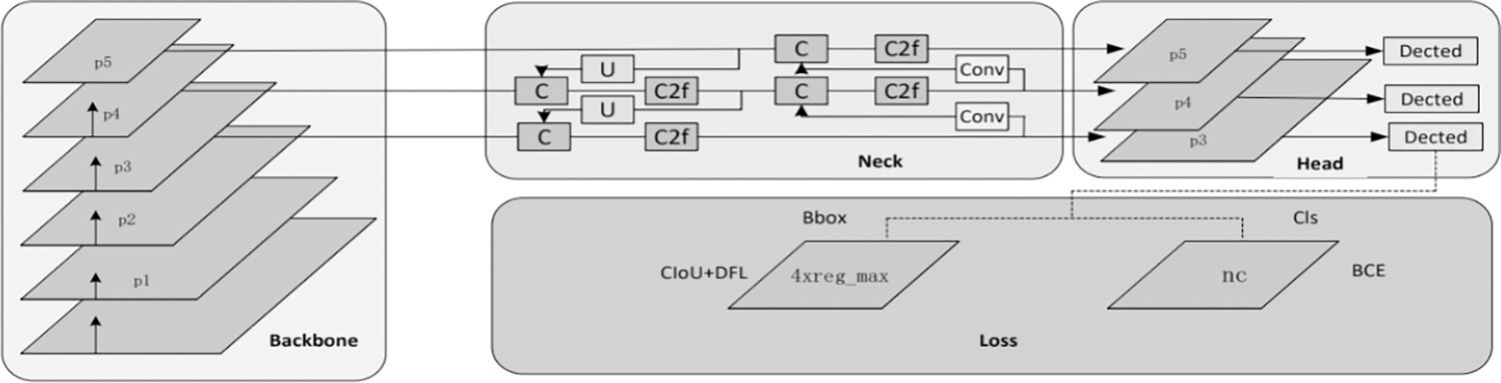
YOLOv8.

A key feature of YOLOv8 is its scalability. It is designed as a framework that supports all previous versions of YOLO, making it easy to switch between different versions and compare their performance. Apart from scalability, YOLOv8 incorporates several innovations that make it widely applicable to object detection and image segmentation tasks, including a new backbone network, new anchor-free network detection heads, and new loss functions. YOLOv8 is also highly efficient, capable of running on both CPUs and GPUs. The backbone of YOLOv8 is similar to YOLOv5, based on the CSP (Cross-Stage-Partial) concept, replacing the C3 module with the C2f module. The C2f module combines C3 and ELAN, following the approach introduced in YOLOv7. This modification allows YOLOv8 to obtain richer gradient flow information while keeping its overall lightweight design. At the end of the backbone, it still employs the popular SPPF (Spatial Pyramid Pooling Fast) module, which passes through three 5x5 Maxpools in sequence and concatenates each layer. This ensures both accuracy at different scales and lightweight object detection. In the neck section, YOLOv8 continues to use the PAN-FPN (Path Aggregation Network-Feature Pyramid Network) feature fusion method to enhance the fusion and utilization of information from different-scale feature layers. YOLOv8’s architecture employs two upsampling layers, multiple C2f modules, and a final decoupled head structure to form the neck module. The idea of head decoupling introduced in YOLOx [48] is utilized for the final part of the neck section. It combines confidence and regression boxes, achieving a new level of accuracy. For positive and negative sample allocation, the YOLOv8 method employs the TOOD (Task-Agnostic Object Detection) task-aligned allocator, which selects positive samples based on the weighted scores of classification and regression. The network architecture of YOLOv8 is depicted in Fig 4, and the detailed parameter settings as show in Table 1.

**Table 1:** Detailed parameter settings of used YOLOV8.

Layers	From	N	Module	Arguments
0	-1	1	Conv	[64,3,2]
1	-1	1	Conv	[128,3,2]
2	-1	3	C2f	[128,True]
3	-1	1	Conv	[256,3,2]
4	-1	6	C2f	[256,True]
5	-1	1	Conv	[512,3,2]
6	-1	6	C2f	[512,True]
7	-1	1	Conv	[1024,3,2]
8	-1	3	C2f	[1024,True]
9	-1	1	SPFF	[1024,5]
10	-1	1	Upsample	[‘None’,2,‘nearest’]
11	[–1,6]	1	Concat	[1]
12	-1	3	C2f	[512]
13	-1	1	Upsample	[‘None’,2,‘nearest’]
14	[–1,4]	1	Concat	[1]
15	-1	3	C2f	[256]
16	-1	1	Conv	[256,3,2]
17	[–1,12]	1	Concat	[1]
18	-1	3	C2f	[512]
19	-1	1	Conv	[512,3,2]
20	[–1,9]	1	Concat	[1]
21	-1	3	C2f	[1024]
22	[15,18,21]	1	Detect	[1]

### 3.2 Cross Progressive Multi-Resolution Representation Integration (CPMRI) model

As the posture recognition task involves pixel-wise key point estimation, it is essential to utilize both low-level and high-level features for locating key points at various scales. High-level features are beneficial for locating key points at larger scales, while low-level features are crucial for accurately estimating key points at smaller scales. To address the challenge of significant scale variations in dance motion skeleton joints, we introduce a cross progressive multi-resolution representation integration model to enhance the robustness of posture recognition against scale variations.

#### 3.2.1. HRNet

**Fig 5:**
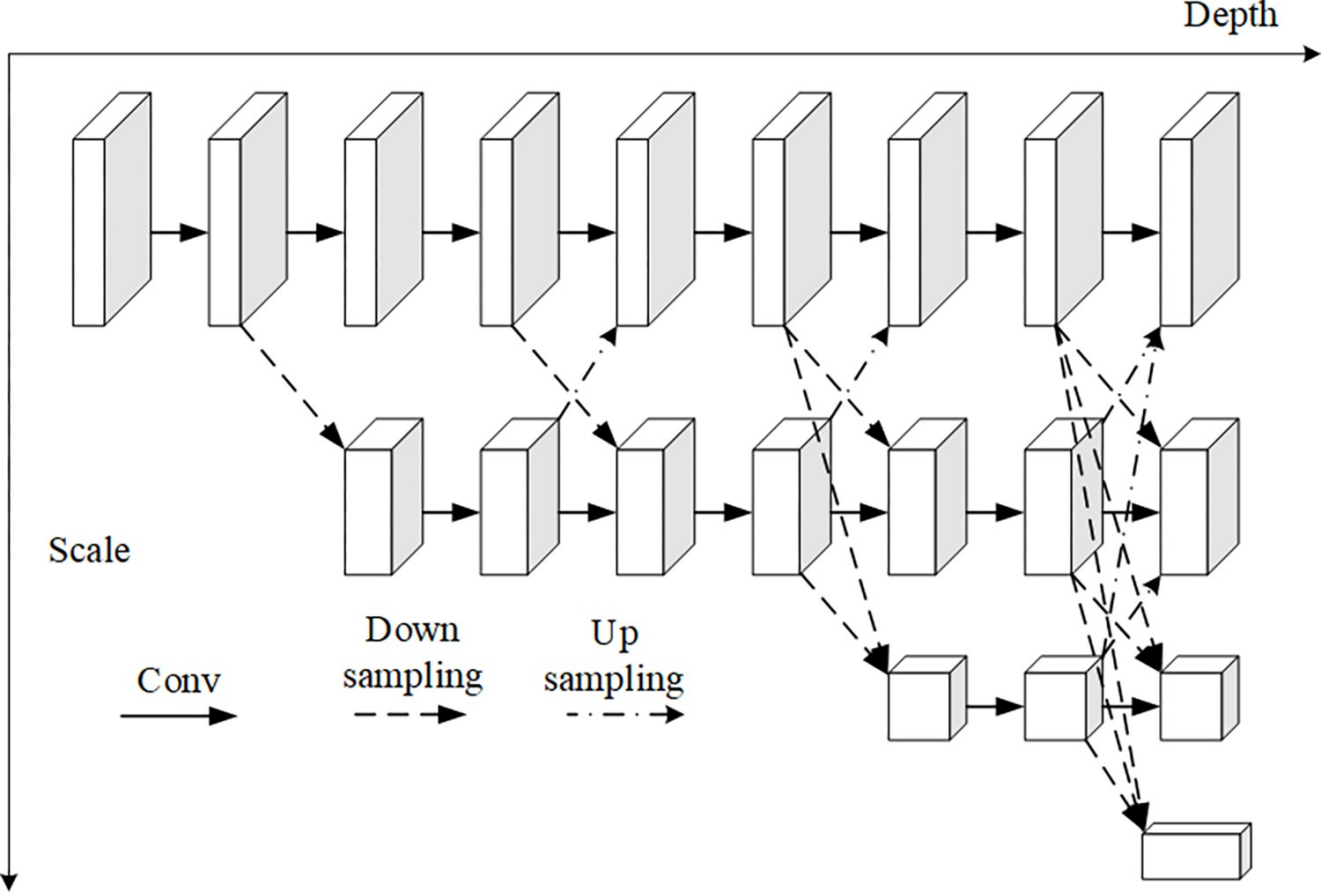
HRNET model.

This paper adopts the HRNet [4] as the backbone network, as illustrated in Fig 5. The HRNet consists of four parallel sub-networks, with each sub-network following the ResNet module design principles, comprising four residual units. The HRNet network excels at extracting multi-resolution features from input images, offering strong representation capabilities. It has achieved remarkable results in various tasks, including object detection, recognition, image segmentation, and human key point estimation. However, in the context of human key point estimation, HRNet does not fully leverage the multi-resolution features it extracts. It only employs the high-resolution features for estimating key point heatmaps and discards the other middle and low-resolution features, resulting in information loss during the feature representation process and impacting the accuracy of key point estimation. Therefore, to address the aforementioned issue, this paper proposes constructing a CPMRI module to enhance the feature representation capabilities in posture recognition.

#### 3.2.2. CPMRI module

**Fig 6:**
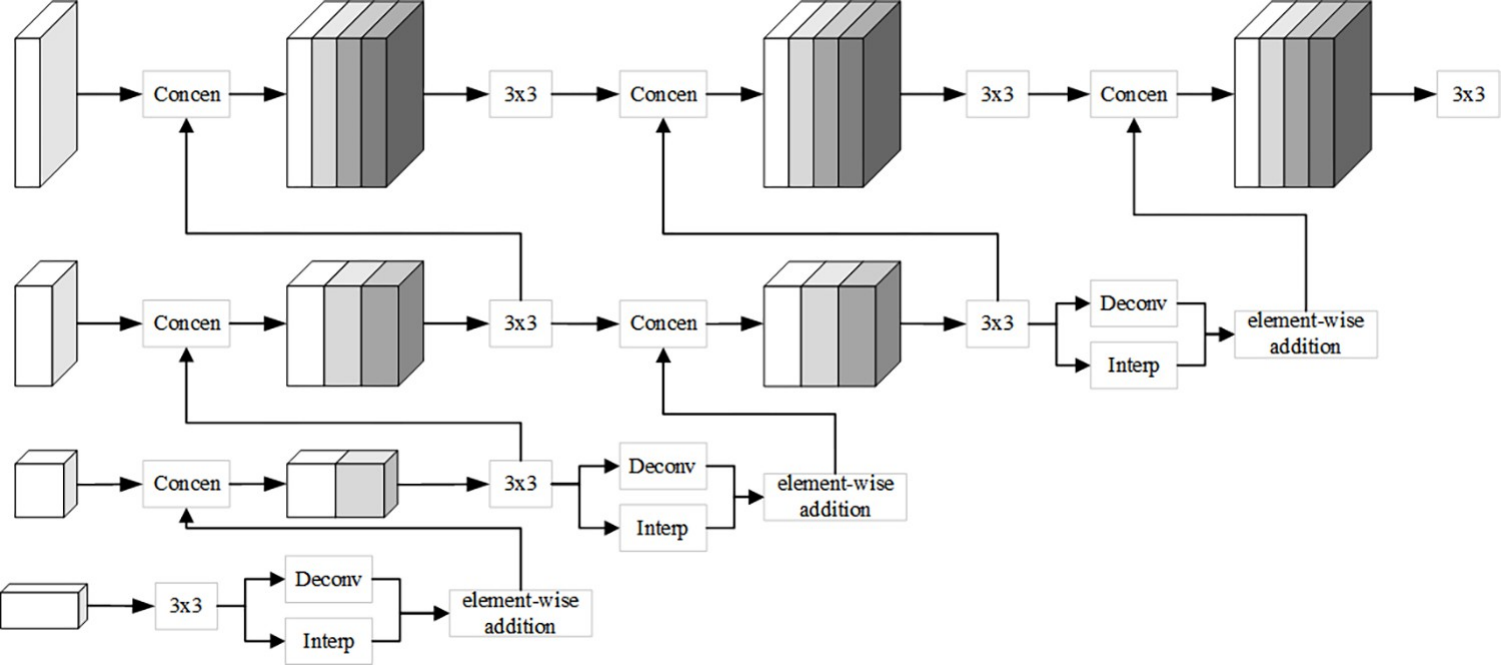
CPMRI module.

In feature representation, low-resolution high-level features contain rich semantic information but relatively coarse positional information, while high-resolution low-level features, although weaker in semantic content, provide accurate positional information. Therefore, this paper proposes a CPMRI module to sequentially fuse high and low-resolution features, enhancing the network’s feature representation capability. As shown in Fig 6, the proposed CPMRI method processes the four different resolution feature maps output by the last aggregation unit of the HRNet network using convolution, interpolation, and deconvolution operations from high-resolution to low-resolution in a cross progressive manner.

In this paper, the four feature maps output by the last aggregation unit of the HRNet network are used as the input features **X** = (**X**_1_,**X**_2_,…**X**_*m*_) for the CPMRI module, where *m* represents the number of corresponding resolutions of the input features (in this paper, *m* = 4). The HRNet-W32 backbone network is employed, with channel numbers of multi-resolution features in its four parallel sub-networks being 32, 6, 4, 128, and 256, respectively. For any feature of the *i*-th resolution, a 3×3 convolution operation is first performed, followed by interpolation and deconvolution operations to upsample the *i*-th resolution feature **X**_*i*−1_ to the modified (*i*-1)-th resolution feature ${\hat{X}}_{i-1}$:

$${\hat{X}}_{i-1}=Int(conv(X_{i}))+Dec(conv(X_{i}))$$ (1)

In which, *conv* represents convolution operation, *Int* and *Dec* represent interpolation and deconvolution operations.

Next, the modified (*i*-1)-th resolution feature ${\hat{X}}_{i-1}$ obtained through upsampling is concatenated with the (*i*-1)-th resolution feature **X**_*i*−1_, resulting in the fused (*i*-1)-th resolution representation ${\bar{X}}_{i-1}$:

$${\bar{X}}_{i-1}=concat({\hat{X}}_{i-1},X_{i-1})$$ (2)

Where *concat* denotes feature concatenation of ${\hat{X}}_{i-1}$ and **X**_*i*−1_.

### 3.3 Tiered Posture Recognition (TPR) module using joint geometric relationships

Given the significant distortions and severe obstructions in dance postures, this paper utilizes the skeleton joints estimated in previous section to predict the joints. By analyzing the geometrical relations between the joints, a TPR module is constructed for multi-level joint estimation, enhancing the accuracy of dancer’s body joint posture recognition.

**Fig 7:**
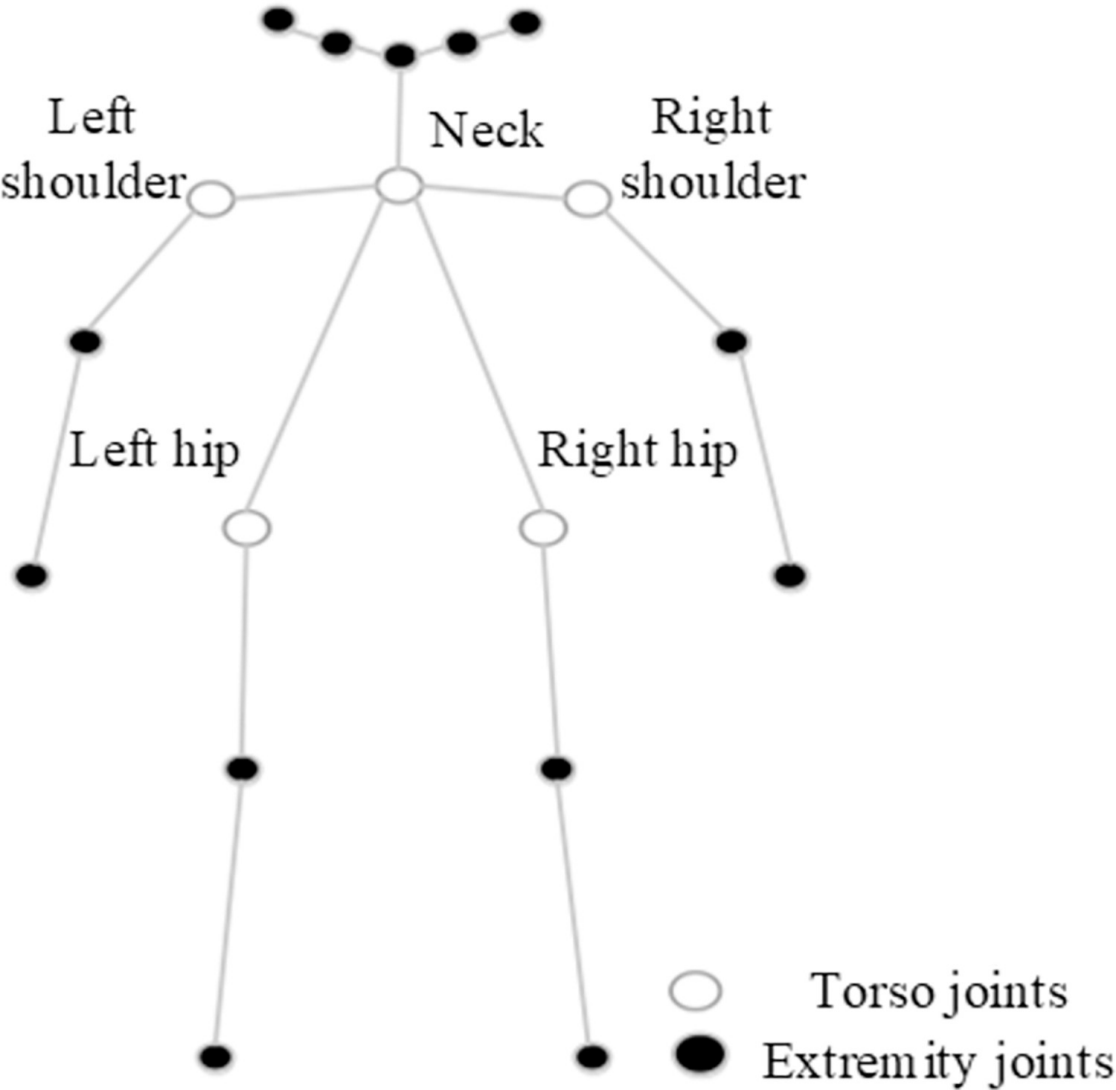
Geometrically-based keypoint segmentation.

To begin with, based on the human body structure, the joints estimated in in previous section are categorized into two types: the first type includes torso joints (*j^torso^*) that connect different parts of the body with small distortions, such as shoulders, hips, and neck. The second type comprises extremity joints (*j^extremity^*) with noticeable distortions, including wrist, elbow, knee, and ankle joints. Subsequently, considering these two types of joints, the TPR model is designed to aggregate all body joints into five parts, namely neck, left shoulder, right shoulder, left hip, and right hip, as shown in Fig 7. These parts are used for joint prediction based on geometrical relations.

As depicted in Fig 1, the designed cross progressive network consists of three tiers.

In the first tier, the CPMRI module designed in previous section predicts heatmaps for all body joints and calculates the corresponding coordinate positions.

Subsequently, the heatmaps obtained in the first tier serve as input for the second tier of the network. Given the characteristics of the relatively stable distortions of torso joints and the significant distortions of extremity joints, the CPMRI module is used to predict the torso joints, *j^torso^,* primarily. This further divides body joints into five parts mainly led by torso joints, also known as five classes (neck, left shoulder, right shoulder, left hip, right hip).

The third tier of the network is constructed by taking all the joints from the first tier and the predicted five classes of torso joints from the tier stage as input. Simultaneously, considering the geometric correlations of the human body’s structure, the network assigns all body joints to the five classes of torso joints based on intra-class relationships, thus establishing connections between extremity joints and torso joints.

Since each category of torso joint can have multiple candidate extremity joints connected to it, and likewise, each extremity joint can potentially be connected to any category of torso joint. Therefore, for any given category of torso joint and extremity joint, let *N*_1_ and *N*_2_ be the sets of candidate nodes for torso joint $j_{1}^{torso}$ and extremity joint $j_{2}^{extremity}$ in part-*c*, respectively. The optimal matching problem for all candidate node intra-category connections can be defined as:

$$\begin{array}{l} Z_{c}=\{z_{j_{1},j_{2}}^{m,n}:j_{1},j_{2}\in \{1,2,\ldots ,J\},m\in \{1,2,\ldots ,N_{1}\},\\ n\in \{1,2,\ldots ,N_{2}\},c\in \{1,2,\ldots ,5\}\} \end{array}$$ (3)

For any pair of connected joints (*j*_1_, *j*_2_), the problem of matching the connections between joints is transformed into a bipartite graph matching subproblem, as per the shared common node approach in the graphical model [49]. By solving the optimal matching problem for all intra-category candidate joint connection sets, the optimal connection between torso joints and extremity joints is obtained, represented as:

$$z_{j_{1},j_{2}}=\max E_{mn}z_{j_{1},j_{2}}^{m,n}$$ (4)

$$s.t.\sum_{N_{1}}z_{j_{1},j_{2}}^{m,n}\leq 1,\sum_{N_{2}}z_{j_{1},j_{2}}^{m,n}\leq 1$$ (5)

In which, *E_mn_* is computed using the Part Affinity Fields for Part Association method [5] to calculate the association probabilities between the joints.

Finally, the optimal matches connecting all torso joints and extremity joints form the final estimation of the body’s posture.

### 3.4 Tiered loss function

Our tiered loss function consists of three components: the difference between all estimated key points from the first tier of the network and the ground truth, the difference between the estimated torso joints from the second tier of the network and the ground truth, and the difference between the estimated extremity joints from the third tier of the network and the ground truth. As shown in (6):

$$Tier\_loss=\frac{1}{3m^{\prime }n^{\prime }}\sum_{i=1}^{n^{\prime }}\sum_{k=1}^{m^{\prime }}(||H(j_{ik})-GT(j_{ik})|{|}^{2}+||H(j_{ik}^{torso})-GT(j_{ik}^{torso})|{|}^{2}+||H(j_{ik}^{extremity})-GT(j_{ik}^{extremity})|{|}^{2})$$ (6)

In which, *n’* represents the number of training samples, and *m’* represents the number of key points. *H*(*j_ik_*) represents the heatmap for the *j*-th key point of the *i*-th sample in the prediction, with the corresponding ground truth denoted as *GT*(*j_ik_*).

## 4. Experiments and results

### 4.1 Dataset

To validate the effectiveness of the proposed method, the experiments conducted in this study include comparative analysis of single-person and multi-person dance posture recognition on both the publicly available MSCOCO2017 dataset [50] and the dance dataset (comprising custom dance data and dance data downloaded from the internet). The MSCOCO2017 dataset consists of 57,000 training data samples (COCO train2017), 5,000 validation data samples (COCO val2017), and 20,000 test data samples (COCO test-dev2017). The custom dance dataset comprises 20,000 dance posture samples, with 10,000 allocated for training and 10,000 for testing purposes.

### 4.2 Evaluation metrics

To evaluate the effectiveness of the proposed method, we employ the OKS (Object Keypoint Similarity)-based Average Precision (AP), mean Average Precision (mAP), and Average Recall (AR) as evaluation metrics.

1) OKS: In the task of human key point evaluation, precision of the key points obtained by the network is not calculated solely based on Euclidean distance. It incorporates new scale information during calculation, resulting in a distance between two key points based on the calculation results. The specific calculation of the OKS metric is as follows:

$$OKS_{p}=\frac{\sum_{i}\exp\{-d_{p_{i}}^{2}/2S_{p}^{2}\sigma_{i}^{2}\}\delta (v_{p_{i}}>0)}{\sum_{i}\delta (v_{p_{i}}>0)}$$ (7)

Where, *p* represents the id of a person in the ground truth, *p_i_* represents the *i*-th key point of the *p*-th person, *d_pi_* represents the Euclidean distance between the detected key point *i* and the *i*-th key point of the *p*-th person in the correct annotations, *S_p_* represents the scale factor of the *p*-th person in the correct annotations, *σ_i_* represents the normalization factor of the *i*-th keypoint, *v_pi_* represents the visibility of the keypoint. *v_pi_* = 1 indicates that the key point is visible and labeled, while *v_pi_* = 2 indicates that the key point is occluded but labeled.

2) AP: In the posture recognition task, both the AP and mAP metrics are calculated based on the OKS metric. For single-person recognition, the AP is calculated as follows:

$$AP=\frac{\sum_{p}\delta (OKS_{p}>T)}{\sum_{p}1}$$ (8)

If a threshold value *T* is given, the calculated result can be obtained through the OKS of all images. For multi-person top-down recognition, the AP calculation remains the same as that of single-person recognition, while for multi-person bottom-up recognition, the AP is calculated as follows:

$$AP=\frac{\sum_{m}\sum_{p}\delta (OKS_{p}>T)}{\sum_{m}\sum_{p}1}$$ (9)

In calculation, *M* represents the number of persons in a given image, and *N* represents the number of persons predicted. As there is no one-to-one correspondence between the predicted *N* persons and the correct *M* persons, we need to calculate the OKS of each person in the correct annotations with the OKS of the *N* predicted persons. This results in an *M*×*N* matrix where each row takes the maximum OKS value as the current OKS. Finally, for each person, there is a scalar OKS, and by setting a threshold T, the AP is calculated for all persons in all images.

AP50 and AP75 represent the AP obtained at intersection over union (IoU) thresholds of 0.50 and 0.75, respectively. APM and APL represent the AP obtained for medium-sized and large-sized targets. Medium-sized targets refer to those with an area greater than 322 but less than 962, while large-sized targets have an area greater than 962.

3) mAP: mAP is another common metric in human posture recognition. It involves obtaining multiple AP metrics by varying the manually set threshold *T* and then calculating the average of these AP values.

4) AR: For a specific dataset, the AR metric is calculated as:

$$AR=\frac{TP}{TP+FN}$$ (10)

In which, TP (true positive) represents the correctly estimated samples, and FN (false negative) represents the samples that were missed in the estimation, which were actually correct. AP and AR are also known as precision and recall, and in the same dataset, AP and AR complement each other. Higher AR values indicate lower FN, meaning fewer missed correct samples and higher recall.

### 4.3 Experiment settings

The experiments in this paper were conducted on a server with Ubuntu 18.04 and four NVIDIA 2080Ti GPUs. The experiments utilized the Python programming language and the Tensorflow deep learning framework. The HRNet-W32 network was chosen as the backbone network, and input images were resized to 256×192. Image augmentation techniques were applied, including random flips, rotations within ±45 degrees, and scale variations within ±35%. The network was optimized using the Adam method, with an initial learning rate of 0.001. The learning rate was decayed to 0.00001 between the 100-th and 130-th epochs, and the network was trained for a total of 210 epochs.

To evaluate the proposed dance posture recognition method, it was compared to several SOTA human pose estimation algorithms, including five top-down approaches (CPN, SimpleBaseline, Mask-RCNN, RMPE, HRNet) and four bottom-up approaches (OpenPose, PifPaf, Hourglass, HigherHRNet), they are described in detail in the related work section.

### 4.4 Ablation experiments

To better analyze the model we designed and reveal the performance of each component, we systematically dissect the different parts of the method and perform comparative experiments with the whole method. By removing individual modules from the overall method and conducting experiments comparing them to the method without those modules, we can assess the impact of each module on pose estimation.

Table 2 presents the results of these ablation experiments and highlights the impact of two crucial components of the proposed network: (1) CPMRI module and (2) TPR.

**Table 2:** The results of the ablation experiments.

Models	*α*	*β*	*γ*	*δ*
Backbone	√	√	√	√
CPMRI	×	√	×	√
TPR	×	×	√	√
mAP(%)	74.46	75.21	75.76	76.29

As shown in Table 2, when both important components of the proposed model are used together (i.e., Model *δ*), the method achieves the highest mAP value. It results in a 1.83% (76.29–74.46) mAP improvement in key point localization accuracy compared to the HRNet-W32 baseline heatmap regression model. In the ablation experiments, Model *β* and Model *γ*, which only add the proposed CPMRI and TPR modules on top of the HRNet-W32 model, respectively, achieve mAP improvements of 0.75% (75.21–74.46) and 1.3% (75.76–74.46). From Table 2, it is evident that the CPMRI module in Model *β* enhances multi-scale feature representation by fusing the multi-resolution features from the HRNet network in a cross progressive way, which benefits key point estimation. Model *γ* incorporates the TPR module, which designs a three-tier tiered network model based on the geometrical relations between key points. This flexible approach handles different categories of torso and extremity key points, solves the optimal matching problem of candidate key points connections within categories, and improves posture recognition accuracy by reasoning about occluded keypoints.

**Fig 8:**
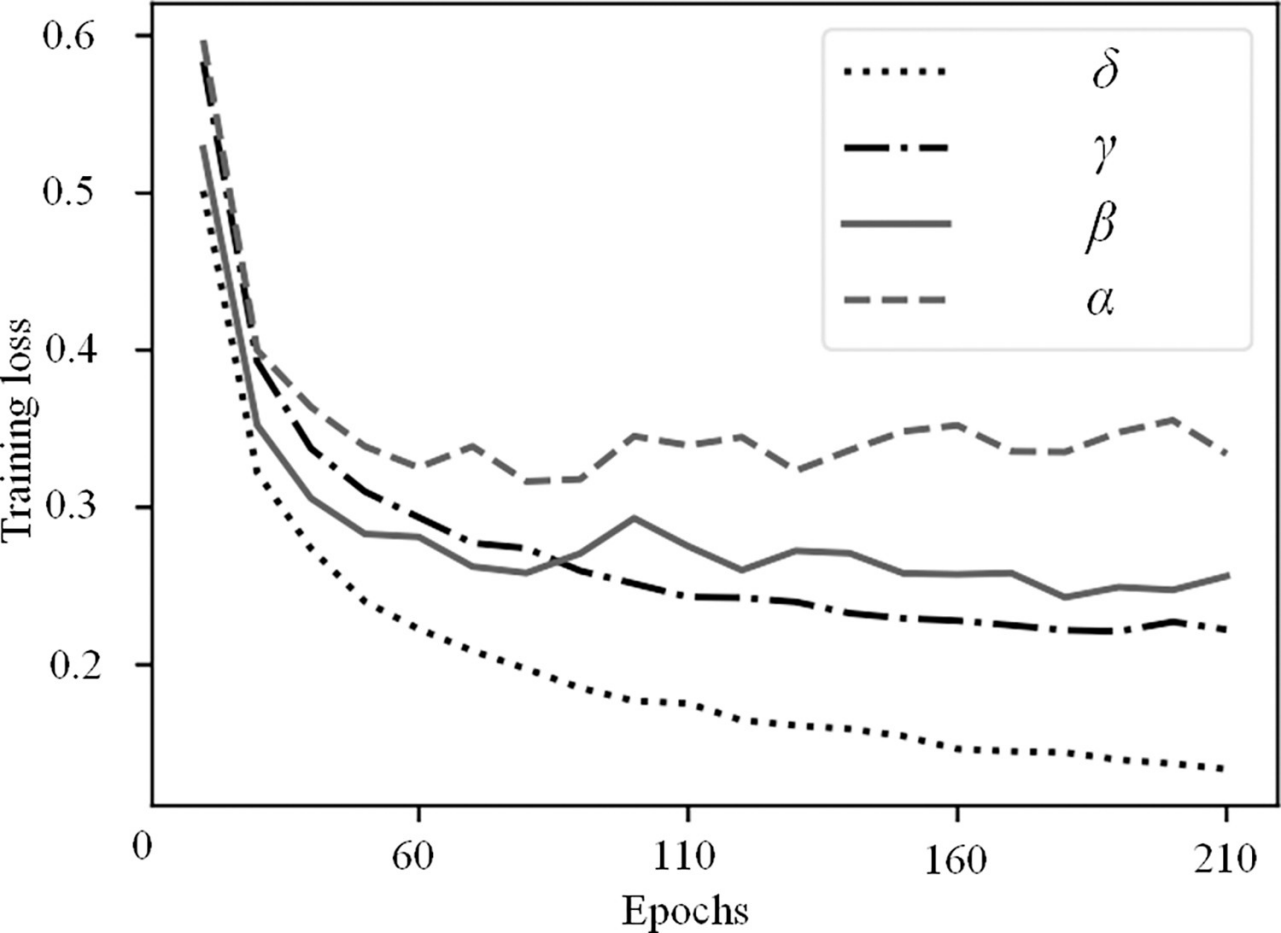
The training curve of the different ablation models.

**Fig 9:**
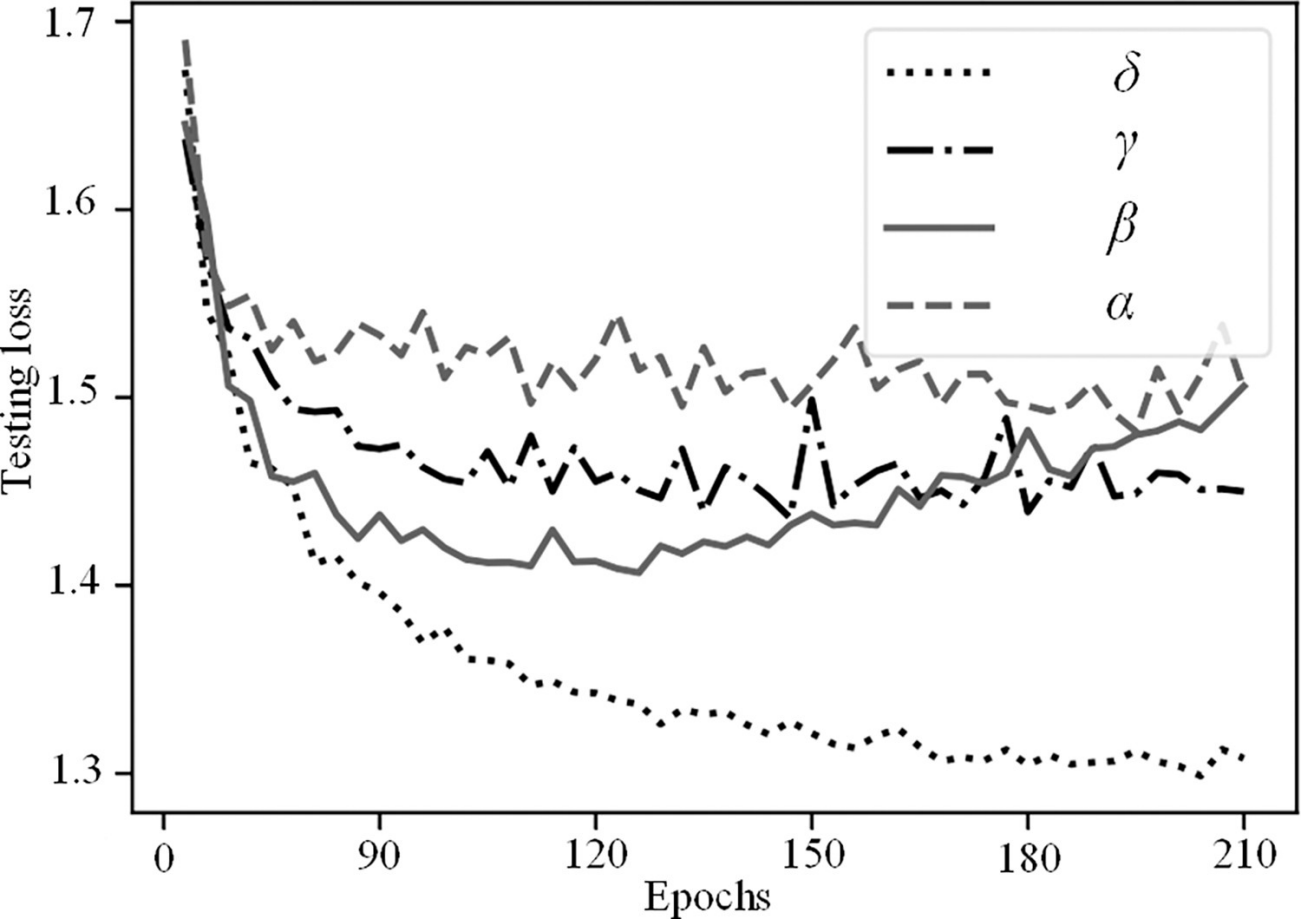
The testing curve of the different ablation models.

Figs 8 and 9 respectively show the training and testing curves of different ablation models. It can be observed that the overall convergence speed follows the order of *δ* > *γ* > *β* > *α*, aligning with the mAP ranking presented in Table 2. Notably, the testing loss curves of models *α*, *β*, and *γ* initially decrease and then exhibit a slight increase, suggesting the possibility of over-fitting. This implies that these models may perform too well on the training data, to the extent of learning noise and non-representative features, thereby diminishing their generalization ability on unseen test data. In contrast, our comprehensive model *δ* demonstrates a consistent downward trend in both training and testing loss curves, indicating the proposed model’s robust generalization capability.

### 4.5 Contrast experiments

#### 4.5.1. MSCOCO2017 dataset

**Fig 10:**
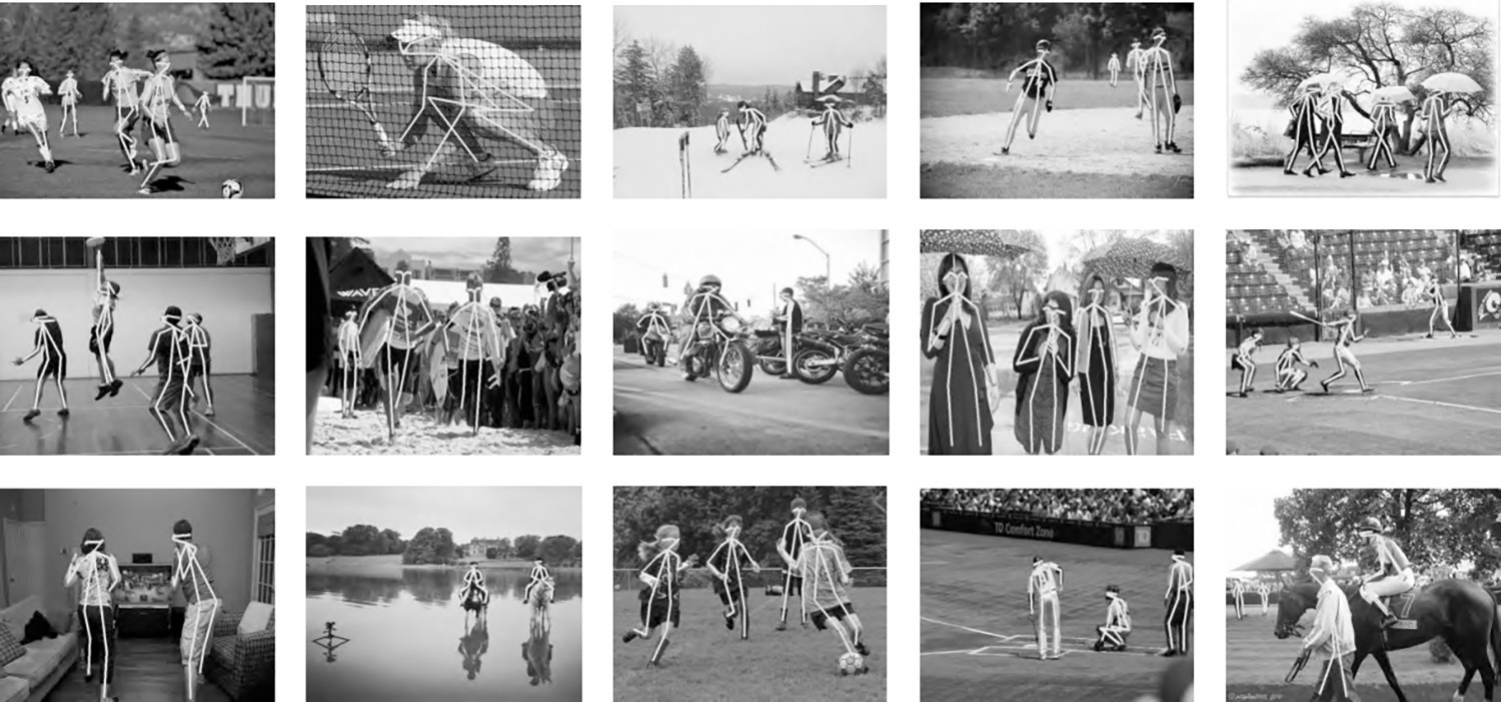
Visualized results in the MSCOCO2017 dataset.

We first conducted a multi-person posture recognition analysis on the COCO test-dev 2017 dataset, comparing the proposed method with nine mainstream methods. To ensure a fair comparison with other methods, we utilized the object detector mentioned in [2] to extract human bounding boxes from the COCO dataset. After obtaining these human detection boxes, we performed human posture recognition using the proposed method. The experimental results are presented in Table 3, and visualizations of our method’s results on the COCO test-dev 2017 dataset are shown in Fig 10.

**Table 3:** The results of the contarst experiments. Underlined denotes SOTA top-down approaches, italicized denotes SOTA bottom-up methods, and bold represents the best result in each metric.

Models	Backbone	Input size	AP	AP50	AP75	APM	APL	AR
CPN	ResNet-Inception	384 × 288	72.1	91.4	80	68.7	77.2	78.5
SimpleBaseline	ResNet-152	384 × 288	73.7	91.9	81.1	70.3	80.0	79.0
Mask-RCNN	ResNet-50-FPN	N/A	63.1	87.3	68.7	57.8	71.4	N/A
RMPE	PyraNet	320 ×256	72.3	89.2	79.1	68.0	78.6	N/A
HRNet	HRNet-W32	256 × 192	74.4	90.5	81.9	70.8	81.0	**81.4**
IFC	BlazePose	384 × 288	70.5	89.2	79.0	68.3	79.6	78.1
Adversarial method	Hourglass-8	256 × 192	73.1	90.2	81.5	71.2	80.8	79.4
*OpenPose*	N/A	N/A	61.8	84.9	67.5	57.1	68.2	66.5
*PifPaf*	N/A	N/A	66.7	N/A	N/A	62.4	72.9	N/A
*Hourglass*	Hourglass	512× 512	65.5	86.8	72.3	60.6	72.6	70.2
*HigherHRNet*	HRNet-W48	640× 640	70.5	89.3	77.2	66.6	75.8	74.9
*MRNet*	MRNet	320× 320	73.0	90.1	80.4	70.6	80.0	78.8
*Dual anatomical approach*	HRNet-W48	640× 640	72.4	88.5	79.1	68.4	79.8	75.3
**Our method**	HRNet-W32	256 × 192	**75.7**	**92.8**	**82.8**	**71.9**	**81.4**	79.7

From Table 3, we can observe that the proposed method achieved a 75.7 AP on the COCO test-dev 2017 dataset when the input image size was set to 256 × 192, with HRNet-W32 as the backbone network. In comparison, the single HRNet-W32 achieved a 74.4 AP, SimpleBaseline achieved 73.7 AP, and Mask-RCNN achieved 63.1 AP. As shown in Table 3, the proposed method outperformed five bottom-up methods, surpassing Openpose, Hourglass, Pifpaf, HigherHRNet-W48, and MRNet by 13.9, 10.2, 9, 5.2, and 2.7 points in terms of AP, respectively. When compared to six top-down methods, the proposed method outperformed Mask-RCNN, CPN, RM-PE, SimpleBaseline, HRNet-W32, and IFC by 12.6, 3.6, 3.4, 2.0, 1.3, and 5.2 points in terms of AP, respectively. In summary, our method achieved favorable multi-person posture recognition results across most evaluation metrics. This success can be attributed to our method’s focus on addressing issues related to variations in human body scale. We introduced a cross progressive multi-resolution representation integration module to enhance the method’s robustness to scale variations. Additionally, to tackle problems related to obstruction and pose distortions, we designed a tiered posture recognition module based on the geometrical relations between key points, which improved the effectiveness of the posture recognition algorithm. As a result, our method excels in scenarios with obstruction, significant scale variations, and complex backgrounds, producing impressive posture recognition results, as illustrated in Fig 10.

#### 4.5.2.The custom dataset

**Fig 11:**
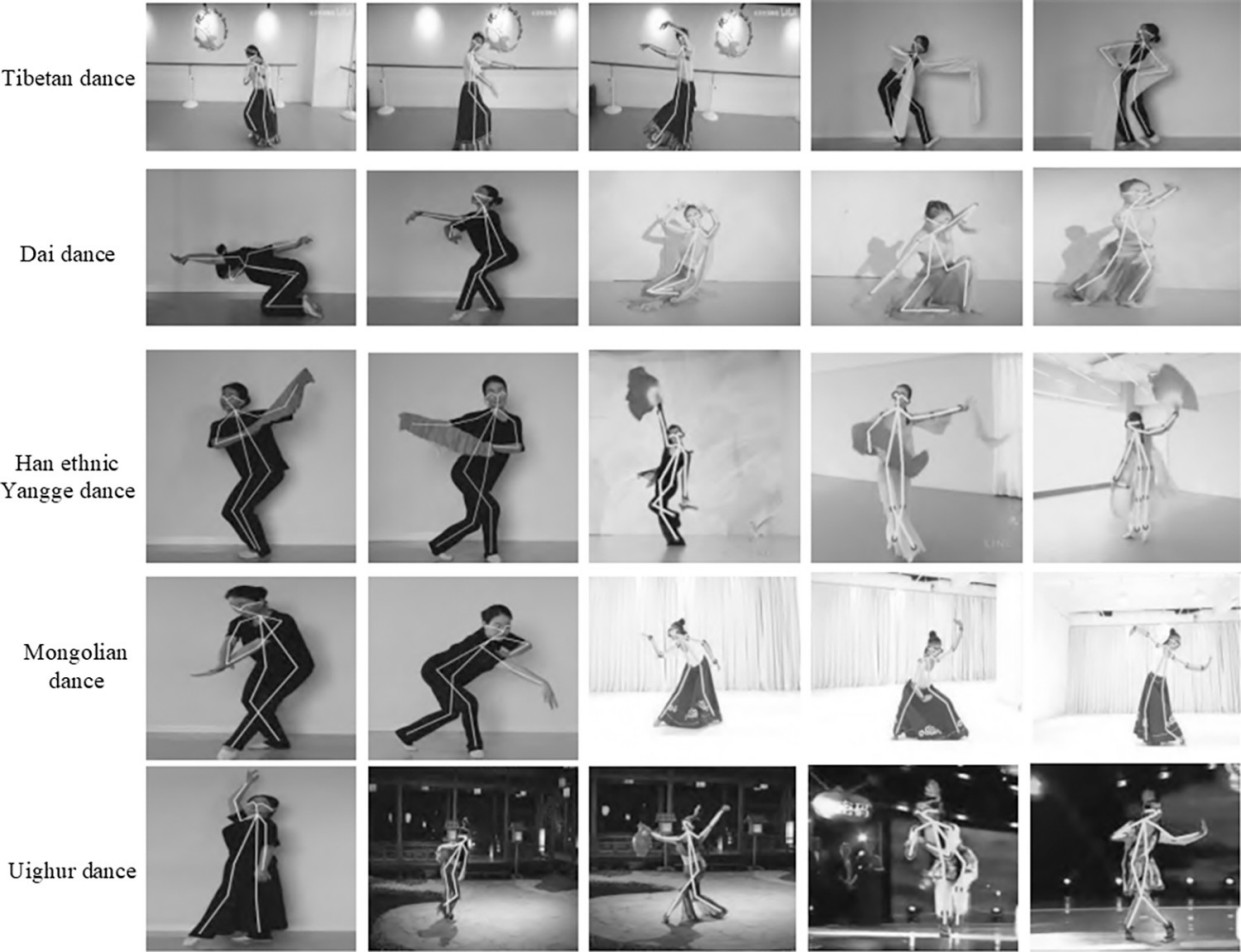
Visualized single-person results in the custom dataset.

**Fig 12:**
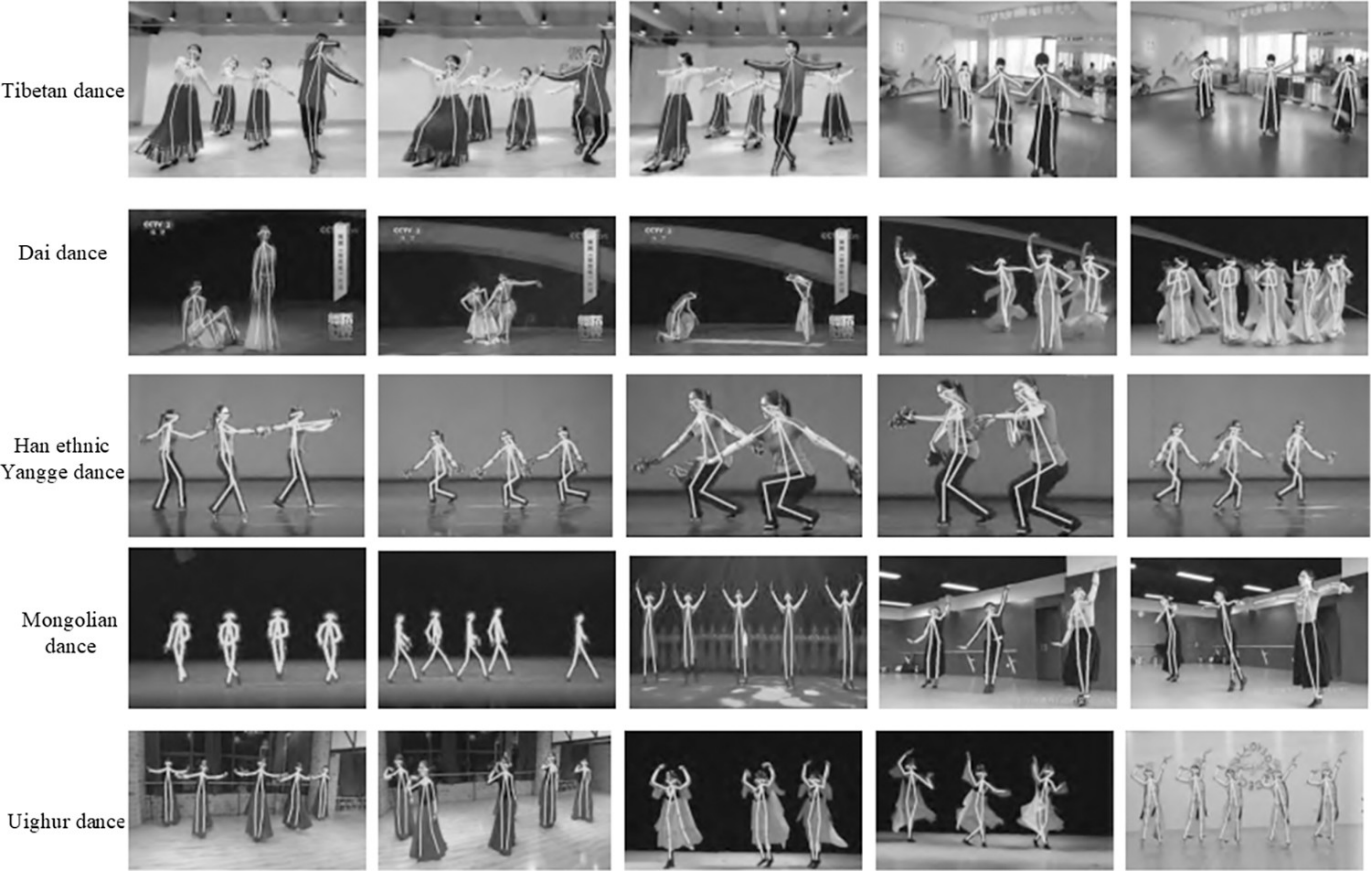
Visualized multi-person results in the custom dataset.

To validate the universality of the proposed method, we conducted human posture recognition on a self-built dance dataset. Since our custom dataset lacks ground truth annotations, quantitative comparisons with other methods were not feasible. Therefore, in this context, we relied on qualitative analysis. Some of the experimental results are shown in Figs 11 and 12.

In Fig 11, we present visual results of our method on five distinct single and single-person ethnic dance datasets, including Tibetan dance, Dai dance, Han ethnic Yangge dance, Mongolian dance, and Uighur dance. These dance forms have unique characteristics, and our qualitative analysis demonstrates the effectiveness of our method. Specifically, in the Tibetan dance, the presence of long skirts worn by the dancers causes obstruction, making it challenging to accurately locate leg joint positions, even for the human eye. Moreover, the sleeves worn by the dancers further obscure arm and leg joints, increasing the difficulty of posture recognition. In such scenarios, our method utilizes geometrical relations between body joints to solve the bipartite graph matching problem through the tiered posture recognition module, thereby predicting occluded key point positions and obtaining more accurate results. In addition, Dai dance exhibits severe pose distortions, self-obstruction, and attire-related obstruction, which pose challenges for key point estimation. In the Han ethnic Yangge dance, as shown in Fig 11, props such as fans significantly obstruct the estimation of dancer poses. Furthermore, rapid actions by the dancers can result in motion blur, making posture recognition more challenging. In Mongolian dance, the poses of the dancers are severely affected by self-obstruction and attire-related obstruction, making it difficult to accurately locate leg joint positions even for human observers. Lastly, in Uighur dance, the effects of lighting, attire, rapid actions, and complex dynamics make dancer key point estimation challenging.

In summary, our method is designed to address challenges such as complex and varied dance actions and severe pose distortions. We have developed a cross progressive multi-resolution representation integration module to enhance the representation of posture recognition features. Simultaneously, we have conducted an analysis of the geometrical relations among human body key points and designed a tiered posture recognition module to infer occluded key points. As illustrated in Fig 11, the proposed method consistently demonstrates its effectiveness in estimating dancer poses under challenging conditions, including scenarios with obstructions, substantial pose distortions, issues caused by varying lighting conditions, and rapid actions. This substantiates the efficacy of the approach presented in this paper.

Fig 12 displays partial visual results of our method on five multi-person ethnic dance datasets. Multi-person dance posture recognition poses a greater challenge than single-person posture recognition, as it involves issues like changes in dancer attire, complex backgrounds, self-obstruction, variations in viewpoint, and the unknown number of people involved. Specifically, in the Tibetan dance dataset, issues include obstruction due to long skirts, changes in camera viewpoints, and rapid actions, making it difficult to accurately locate the body key points of multiple dancers simultaneously. The Dai dance dataset presents challenges due to dim stage lighting, complex and varied dancer actions, dramatic changes in dancer poses, attire-related interference, mutual obstruction among dancers, and self-obstruction. These factors significantly increase the difficulty of estimating key points for multiple dancers. In the Han ethnic Yangge dance dataset, large variations in pose, complex and varied dancer actions, and changes in camera viewpoints add to the complexity of posture recognition. The Mongolian dance dataset exhibits significant variations in pose scale, severe obstruction, and attire-related obstruction, further complicating key point estimation for multiple dancers. Lastly, the Uighur dance dataset presents challenges related to severe attire-related obstruction, complex changes in dance actions, and other dynamic variations in the poses of multiple dancers.

In summary, our posture recognition method has demonstrated promising results in both standard human pose estimation datasets and self-built single and multi-person dance datasets. However, we acknowledge that this method primarily addresses common characteristics of dance actions, such as complex and dynamic actions, strong pose continuity, and issues related to obstruction. Our future research will delve deeper into the diverse characteristics of dance actions specific to different ethnic groups.

## 5. Conclusion

The CPMRI and TPR modules introduced in this study have notably advanced multi-person dance posture recognition. By integrating high-level and low-level representations through cross progressive fusion and addressing distortions with tiered posture recognition, these modules significantly improve the robustness and accuracy of recognition, especially in challenging real-world scenarios. This approach not only captures the nuances of human poses effectively but also sets a foundation for future advancements. Building upon these successes, future work will focus on enhancing dataset quality, incorporating temporal context in posture recognition, developing lightweight models for broader application, and exploring the potential of unsupervised learning models. These directions promise to further elevate the field of dance posture recognition, expanding its applicability and efficiency. Despite our method’s significant advancements in multi-person dance posture recognition through cross progressive multi-resolution representation integration, challenges persist, including enhancing dataset quality for better model generalization, incorporating temporal information for comprehensive 2D posture analysis, developing lightweight models for edge computing, and leveraging unsupervised learning to improve adaptability and reduce dependency on annotated data.

### Statement

This study involves the collection and analysis of a custom dance dataset comprising 20,000 dance samples, divided equally between training and testing purposes. We hereby confirm that our methods of data collection and analysis are in full compliance with the terms and conditions stipulated by the data source.

## References

[1] Chen Y, Wang Z, Peng Y, et al. Cascaded pyramid network for multi-person pose estimation[C]//Proceedings of the IEEE conference on computer vision and pattern recognition. 2018: 7103–7112.

[2] Fang H S, Xie S, Tai Y W, et al. Rmpe: Regional multi-person pose estimation[C]//Proceedings of the IEEE international conference on computer vision. 2017: 2334–2343.

[3] Newell A, Yang K, Deng J. Stacked hourglass networks for human pose estimation[C]//Computer Vision–ECCV 2016: 14th European Conference, Amsterdam, The Netherlands, October 11–14, 2016, Proceedings, Part VIII 14. Springer International Publishing, 2016: 483–499.

[4] Wang J, Sun K, Cheng T, et al. Deep high-resolution representation learning for visual recognition[J]. IEEE transactions on pattern analysis and machine intelligence, 2020, 43(10): 3349–3364. 10.1109/TPAMI.2020.2983686 32248092

[5] Chen W, Jiang Z, Guo H, et al. Fall detection based on key points of human-skeleton using openpose[J]. Symmetry, 2020, 12(5): 744.

[6] Pishchulin L, Insafutdinov E, Tang S, et al. Deepcut: Joint subset partition and labeling for multi person pose estimation[C]//Proceedings of the IEEE conference on computer vision and pattern recognition. 2016: 4929–4937.

[7] Cheng B, Xiao B, Wang J, et al. Higherhrnet: Scale-aware representation learning for bottom-up human pose estimation[C]//Proceedings of the IEEE/CVF conference on computer vision and pattern recognition. 2020: 5386–5395.

[8] Kreiss S, Bertoni L, Alahi A. Pifpaf: Composite fields for human pose estimation[C]//Proceedings of the IEEE/CVF conference on computer vision and pattern recognition. 2019: 11977–11986.

[9] Leone A, Rescio G, Caroppo A, et al. Human Postures Recognition by Accelerometer Sensor and ML Architecture Integrated in Embedded Platforms: Benchmarking and Performance Evaluation[J]. Sensors, 2023, 23(2): 1039. 36679839 10.3390/s23021039 PMC9865298

[10] Li L, Yang G, Li Y, et al. Abnormal sitting posture recognition based on multi-scale spatiotemporal features of skeleton graph[J]. Engineering Applications of Artificial Intelligence, 2023, 123: 106374.

[11] Zhang H, Tian Y, Zhang Y, et al. Pymaf-x: Towards well-aligned full-body model regression from monocular images[J]. IEEE Transactions on Pattern Analysis and Machine Intelligence, 2023. 10.1109/TPAMI.2023.3271691 37126625

[12] Sapp B, Weiss D, Taskar B. Parsing human motion with stretchable models[C]//CVPR 2011. IEEE, 2011: 1281–1288.

[13] Zuffi S, Freifeld O, Black M J. From pictorial structures to deformable structures[C]//2012 IEEE conference on computer vision and pattern recognition. IEEE, 2012: 3546–3553.

[14] Song W, Zhang X, Guo Y, et al. Automatic Generation of 3D Scene Animation Based on Dynamic Knowledge Graphs and Contextual Encoding[J]. International Journal of Computer Vision, 2023: 1–29.

[15] Xue Y, Bhatnagar B L, Marin R, et al. NSF: Neural Surface Fields for Human Modeling from Monocular Depth[C]//Proceedings of the IEEE/CVF International Conference on Computer Vision. 2023: 15049–15060.

[16] Yuan J, Zhang Y, Wei C, et al. A Fully Self‐Powered Wearable Leg Movement Sensing System for Human Health Monitoring[J]. Advanced Science, 2023, 10(29): 2303114. 37590377 10.1002/advs.202303114 PMC10582417

[17] Aonty S S, Deb K, Sarma M S, et al. Multi-Person Pose Estimation Using Group-Based Convolutional Neural Network Model[J]. IEEE Access, 2023.

[18] Xiao B, Wu H, Wei Y. Simple baselines for human pose estimation and tracking[C]//Proceedings of the European conference on computer vision (ECCV). 2018: 466–481.

[19] Wang J, Long X, Gao Y, et al. Graph-pcnn: Two stage human pose estimation with graph pose refinement[C]//Computer Vision–ECCV 2020: 16th European Conference, Glasgow, UK, August 23–28, 2020, Proceedings, Part XI 16. Springer International Publishing, 2020: 492–508.

[20] Yang Y, Wu J, Li H, et al. Dynamical system inspired adaptive time stepping controller for residual network families[C]//Proceedings of the AAAI Conference on Artificial Intelligence. 2020, 34(04): 6648–6655.

[21] Iqbal U, Gall J. Multi-person pose estimation with local joint-to-person associations[C]//Computer Vision–ECCV 2016 Workshops: Amsterdam, The Netherlands, October 8–10 and 15–16, 2016, Proceedings, Part II 14. Springer International Publishing, 2016: 627–642.

[22] Papandreou G, Zhu T, Kanazawa N, et al. Towards accurate multi-person pose estimation in the wild[C]//Proceedings of the IEEE conference on computer vision and pattern recognition. 2017: 4903–4911.

[23] Su K, Yu D, Xu Z, et al. Multi-person pose estimation with enhanced channel-wise and spatial information[C]//Proceedings of the IEEE/CVF conference on computer vision and pattern recognition. 2019: 5674–5682.

[24] Qiu L, Zhang X, Li Y, et al. Peeking into occluded joints: A novel framework for crowd pose estimation[C]//Computer Vision–ECCV 2020: 16th European Conference, Glasgow, UK, August 23–28, 2020, Proceedings, Part XIX 16. Springer International Publishing, 2020: 488–504.

[25] Rafi U, Leibe B, Gall J, et al. An Efficient Convolutional Network for Human Pose Estimation[C]//BMVC. 2016, 1: 2.

[26] Li Y, Zhang S, Wang Z, et al. Tokenpose: Learning keypoint tokens for human pose estimation[C]//Proceedings of the IEEE/CVF International conference on computer vision. 2021: 11313–11322.

[27] Insafutdinov E, Pishchulin L, Andres B, et al. Deepercut: A deeper, stronger, and faster multi-person pose estimation model[C]//Computer Vision–ECCV 2016: 14th European Conference, Amsterdam, The Netherlands, October 11–14, 2016, Proceedings, Part VI 14. Springer International Publishing, 2016: 34–50.

[28] Osokin D. Real-time 2d multi-person pose estimation on cpu: Lightweight openpose[J]. arXiv preprint arXiv:1811.12004, 2018.

[29] Hidalgo G, Raaj Y, Idrees H, et al. Single-network whole-body pose estimation[C]//Proceedings of the IEEE/CVF international conference on computer vision. 2019: 6982–6991.

[30] Newell A, Huang Z, Deng J. Associative embedding: End-to-end learning for joint detection and grouping[J]. Advances in neural information processing systems, 2017, 30.

[31] Jin S, Liu W, Xie E, et al. Differentiable hierarchical graph grouping for multi-person pose estimation[C]//Computer Vision–ECCV 2020: 16th European Conference, Glasgow, UK, August 23–28, 2020, Proceedings, Part VII 16. Springer International Publishing, 2020: 718–734.

[32] Yu C, Xiao B, Gao C, et al. Lite-hrnet: A lightweight high-resolution network[C]//Proceedings of the IEEE/CVF conference on computer vision and pattern recognition. 2021: 10440–10450.

[33] Papandreou G, Zhu T, Chen L C, et al. Personlab: Person pose estimation and instance segmentation with a bottom-up, part-based, geometric embedding model[C]//Proceedings of the European conference on computer vision (ECCV). 2018: 269–286.

[34] Kocabas M, Karagoz S, Akbas E. Multiposenet: Fast multi-person pose estimation using pose residual network[C]//Proceedings of the European conference on computer vision (ECCV). 2018: 417–433.

[35] Geng Z, Sun K, Xiao B, et al. Bottom-up human pose estimation via disentangled keypoint regression[C]//Proceedings of the IEEE/CVF conference on computer vision and pattern recognition. 2021: 14676–14686.

[36] Wang Y, Li M, Cai H, et al. Lite pose: Efficient architecture design for 2d human pose estimation[C]//Proceedings of the IEEE/CVF Conference on Computer Vision and Pattern Recognition. 2022: 13126–13136.

[37] Nie X, Feng J, Zhang J, et al. Single-stage multi-person pose machines[C]//Proceedings of the IEEE/CVF international conference on computer vision. 2019: 6951–6960.

[38] Tang W, Yu P, Wu Y. Deeply learned compositional models for human pose estimation[C]//Proceedings of the European conference on computer vision (ECCV). 2018: 190–206.

[39] Nie X, Feng J, Xing J, et al. Generative partition networks for multi-person pose estimation[J]. arXiv preprint arXiv:1705.07422, 2017.

[40] https://github.com/ultralytics/ultralytics.

[41] Girshick R. Fast r-cnn[C]//Proceedings of the IEEE international conference on computer vision. 2015: 1440–1448.

[42] Ge Z, Liu S, Wang F, et al. Yolox: Exceeding yolo series in 2021[J]. arXiv preprint arXiv:2107.08430, 2021.

[43] Trudeau R J. Introduction to graph theory[M]. Courier Corporation, 2013.

[44] Luo Y T W C Y, Zhang Y. Humble Teachers Teach Better Students for Semi Supervised Object Detection: Supplementary Materials[J].

[45] Andriluka M, Iqbal U, Insafutdinov E, et al. Posetrack: A benchmark for human pose estimation and tracking[C]//Proceedings of the IEEE conference on computer vision and pattern recognition. 2018: 5167–5176.

[46] Sherstinsky A. Fundamentals of Recurrent Neural Network (RNN) and Long Short-Term Memory (LSTM) Network[J]. Physica D: Nonlinear Phenomena, 404[2023-11-08]. doi: 10.1016/j.physd.2019.132306

[47] Li N, Boers S. Human Motion Recognition in Dance Video Images Based on Attitude Estimation[J]. Wireless Communications and Mobile Computing, 2023, 2023.

[48] Sheu M H, Morsalin S M S, Hsu C C, et al. Improvement of Human Pose Estimation and Processing With the Intensive Feature Consistency Network[J]. IEEE Access, 2023, 11: 28045–28059. doi: 10.1109/ACCESS.2023.3258417

[49] Li H, Yao H, Hou Y. HPnet: Hybrid Parallel Network for Human Pose Estimation[J]. Sensors, 2023, 23(9): 4425. 37177628 10.3390/s23094425 PMC10181615

[50] Andriluka M, Pishchulin L, Gehler P, et al. 2d human pose estimation: New benchmark and state of the art analysis[C]//Proceedings of the IEEE Conference on computer Vision and Pattern Recognition. 2014: 3686–3693.

